# Acute heart failure physiology reversed by endoscopic removal of retained toothbrushes in gastro-cardiac (Roemheld) syndrome: a case report

**DOI:** 10.1093/ehjcr/ytag445

**Published:** 2026-06-09

**Authors:** Ivana Jurin, Tomislava Bodrožić Džakić Poljak, Antonio Bulum, Frane Paštrović

**Affiliations:** Department of Cardiovascular Diseases, University Hospital Dubrava, Avenija Gojka Šuška 6, Zagreb 10000, Croatia; Department of Cardiovascular Diseases, University Hospital Dubrava, Avenija Gojka Šuška 6, Zagreb 10000, Croatia; Department of Diagnostic and Interventional Radiology, University Hospital Dubrava,Avenija Gojka Šuška 6, Zagreb 10000, Croatia; Department of Internal Medicine, University Hospital Dubrava, Avenija Gojka Šuška 6, Zagreb 10000, Croatia

**Keywords:** Case report, Gastro-cardiac syndrome, Roemheld syndrome, Foreign body ingestion, Toothbrush, Cardiovascular magnetic resonance

## Abstract

**Background:**

Roemheld (gastro-cardiac) syndrome describes cardiac symptoms precipitated by gastrointestinal pathology, typically gastric distension or hiatal hernia. Foreign body ingestion is common, but retention of long objects such as toothbrushes is rare and usually requires endoscopic removal. We present an unusual case of acute heart failure with restrictive filling pattern and markedly elevated NT-proBNP, in which cardiac symptoms resolved after extraction of two retained toothbrushes from the stomach.

**Case summary:**

A 44-year-old woman was admitted for progressive dyspnoea, 6-kg weight gain, and NT-proBNP of 853 pg/mL. Echocardiography revealed restrictive diastolic dysfunction. After IV furosemide, diuresis reached 4 L in the first 24 h. Cardiac magnetic resonance showed preserved ejection fraction and no structural abnormalities; however, two foreign bodies were incidentally visualized within the stomach. Gastroscopy was performed and two toothbrushes were removed. Total diuresis reached 12 L and NT-proBNP decreased to 58 pg/mL by Day 3. Psychiatry evaluation excluded psychosis or self-harm intent; the patient denied ingestion. She reported wearing complete dentures due to early onset periodontitis at age 33. Symptoms resolved completely after extraction.

**Conclusion:**

This case supports that gastric retention of foreign bodies may trigger or exacerbate heart failure via gastric distension, vagal stimulation, and impaired venous return—a form of Roemheld gastro-cardiac syndrome.

Learning points

- In unexplained dyspnoea, oedema, and apparent cardiac congestion, extracardiac causes such as gastric distension or retained foreign bodies should remain in the differential diagnosis.
- Cardiac magnetic resonance may reveal an extracardiac trigger, and long rigid gastric foreign bodies such as toothbrushes should undergo urgent endoscopic removal because spontaneous passage is unlikely.

## Introduction

Roemheld syndrome (gastro-cardiac syndrome) refers to cardiac or cardiopulmonary symptoms triggered by gastrointestinal pathology, likely through combined mechanical and autonomic mechanisms.^1–4^ Long rigid objects such as toothbrushes rarely pass the pylorus or duodenal sweep spontaneously, and current endoscopic guidance recommends urgent removal because of the risk of retention and complications.^5,6^ We present a case in which retained toothbrushes were associated with acute heart-failure physiology that reversed rapidly after endoscopic extraction.

## Summary figure

**Table:** 

Time	Key events
Day 0 (admission)	Progressive dyspnoea, abdominal bloating, peripheral oedema, and rapid 6-kg weight gain over the preceding week; *n*-terminal pro-B-type natriuretic peptide (NT-proBNP) 853 pg/mL (upper limit of normal<125 pg/mL); ECG with sinus rhythm, PR 138 ms, QRS 84 ms, QTc 418 ms, and biphasic T waves in V1–V4; TTE interpreted as restrictive filling with elevated filling pressures; intravenous furosemide started.
Day 2	Cardiovascular magnetic resonance: Preserved biventricular systolic function; normal pericardium; no myocardial oedema or late gadolinium enhancement; incidental identification of two linear gastric foreign bodies.
Day 2	Urgent upper gastrointestinal endoscopy: two retained toothbrushes removed using a snare and overtube.
Day 3	Brisk post-extraction diuresis (4 L/24 h; 12 L/72 h cumulative); NT-proBNP 58 pg/mL (upper limit of normal <125 pg/mL); complete resolution of dyspnoea, bloating, and oedema.
1-Month follow-up	NT-proBNP 73 pg/mL (upper limit of normal <125 pg/mL); repeat TTE essentially normal, with LVEF ∼70%, LAVI 26 mL/m^2^, E/e′ 7.22, TAPSE 24 mm, mild TR, and estimated RVSP up to 30 mmHg; patient remained asymptomatic.
3-Month follow-up	Patient remained clinically well; repeat TTE completely normal; NT-proBNP remained within the normal range (<125 pg/mL); she continued to deny swallowing the toothbrushes and remained surprised by their presence.

*Figure 1* is an educational landscape summary figure uploaded separately as a high-resolution image. It illustrates the proposed gastro-cardiac mechanism, the diagnostic pathway, and the rapid post-extraction reversal of congestion.

## Case presentation

A 44-year-old woman was admitted with progressive dyspnoea, abdominal bloating, peripheral oedema, and rapid 6-kg weight gain over the preceding week. On admission, *n*-terminal pro-B-type natriuretic peptide (NT-proBNP) was 853 pg/mL upper limit of normal <125 pg/mL). A 12-lead electrocardiogram showed sinus rhythm at 66 b.p.m. with an intermediate axis, PR interval 138 ms, QRS duration 84 ms, QTc 418 ms, biphasic T-wave morphology in V1–V2, and shallow biphasic T waves extending to V3–V4.

**Figure 1:**
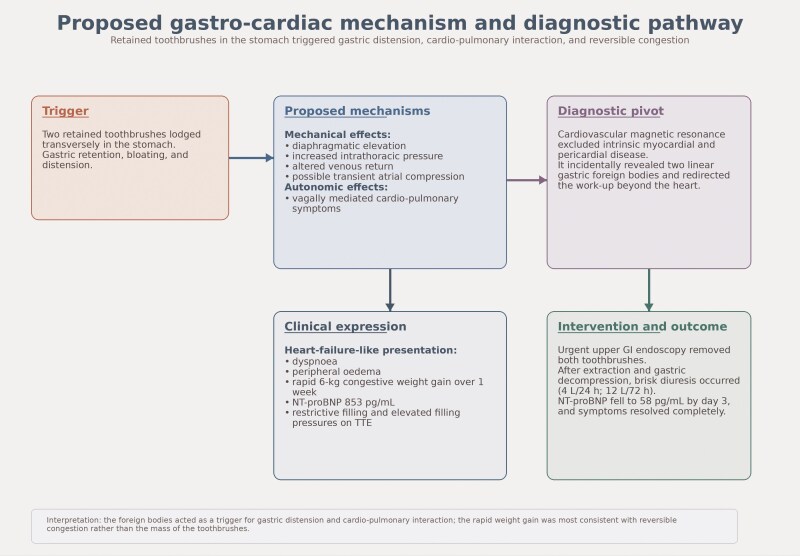
Proposed gastro-cardiac mechanism and diagnostic pathway. Educational summary illustrating how retained toothbrushes may have triggered gastric distension, cardio-pulmonary interaction, and reversible congestion, and how cardiovascular magnetic resonance redirected the diagnostic evaluation towards an extracardiac cause.

Transthoracic echocardiography demonstrated a normal-sized left ventricle (left ventricular end-diastolic diameter 4.0 cm; left ventricular end-systolic diameter 2.6 cm) with normal wall thickness (interventricular septum 1.1 cm; posterior wall 1.1 cm) and preserved systolic function (left ventricular ejection fraction approximately 60%–65%). Left atrial diameter was 3.1 cm, left atrial volume index (LAVI) 26 mL/m^2^, and right ventricular systolic function was preserved (tricuspid annular plane systolic excursion [TAPSE] 24 mm). Diastolic findings were reported as a restrictive filling pattern with elevated filling pressures, supported by E velocity 1.12 m/s, E/A ratio 1.5, average E/e′ 18.23, mild tricuspid regurgitation with TR Vmax 2.72 m/s and estimated right ventricular systolic pressure (RVSP) 34.7 mmHg, and a 19 mm inferior vena cava with only partial inspiratory collapse. There was trivial mitral regurgitation, a tricuspid aortic valve without significant stenosis or regurgitation, and no haemodynamically significant intracardiac shunt or left ventricular outflow tract obstruction. Intravenous furosemide was started for suspected congestion. Representative admission transthoracic echocardiographic images and Doppler/tissue Doppler tracings are shown in *Figure 2A–D*.

**Figure 2:**
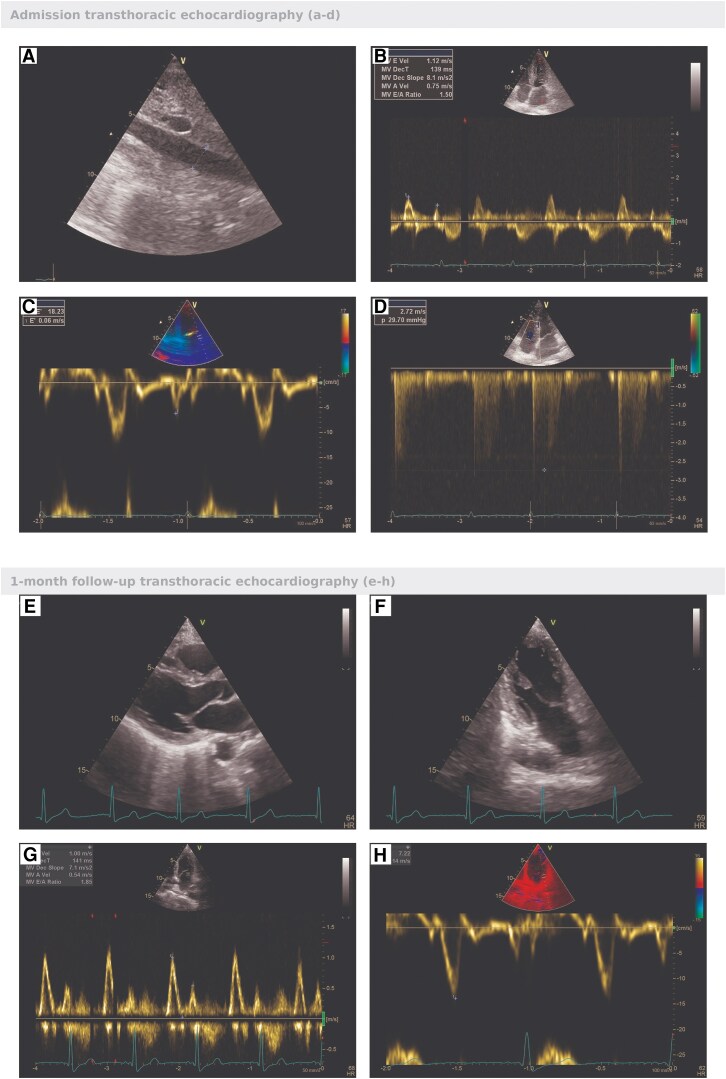
Representative transthoracic echocardiographic images and tracings at presentation and 1-month follow-up. (*A*) Representative two-dimensional admission transthoracic echocardiographic view. (*B*) Admission mitral inflow pulse-wave Doppler demonstrating E velocity 1.12 m/s, A velocity 0.75 m/s, E/A ratio 1.50, and deceleration time 139 ms. (*C*) Admission tissue Doppler tracing demonstrating average E/e′ 18.23. (*D*) Admission continuous-wave Doppler across the tricuspid regurgitation jet demonstrating peak velocity 2.72 m/s. (*E*) One-month follow-up parasternal long-axis view. (*F*) One-month follow-up apical four-chamber view. (*G*) One-month follow-up mitral inflow pulse-wave Doppler demonstrating E velocity 1.0 m/s, A velocity 0.54 m/s, and deceleration time 141 ms. (*H*) One-month follow-up tissue Doppler tracing demonstrating average E/e′ 7.22.

On hospital Day 2, cardiovascular magnetic resonance (CMR) showed preserved biventricular systolic function, a normal pericardium, and no myocardial oedema or late gadolinium enhancement. Importantly, it also incidentally demonstrated linear intragastric foreign-body material adjacent to the heart, subsequently confirmed at endoscopy to represent retained toothbrushes; representative images are shown in *Figure 3A–C*. This redirected the diagnostic work-up beyond primary cardiac disease.

**Figure 3:**
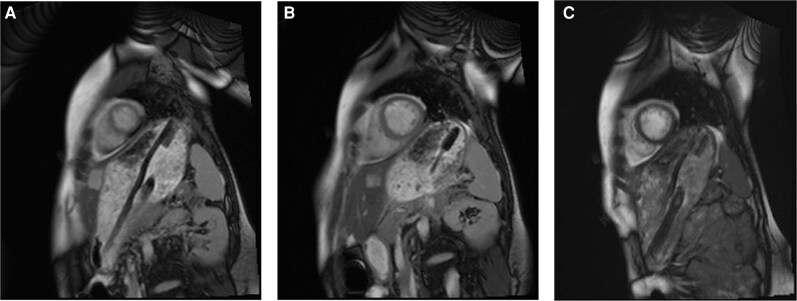
Representative cardiovascular magnetic resonance cine images showing linear intragastric foreign-body material adjacent to the heart. These findings prompted urgent upper gastrointestinal endoscopy and were later confirmed to represent retained toothbrushes.

Urgent upper gastrointestinal endoscopy performed the same day demonstrated retained toothbrushes within the stomach; both were removed using a retrieval snare and overtube (*Figure 4A and B*). Following extraction and gastric decompression, alongside continued intravenous diuretic therapy, urine output increased markedly (4000 mL in the first 24 h; 12 L cumulatively over 72 h). NT-proBNP fell to 58 pg/mL ( limit of normal <125 pg/mL) by Day 3 and the dyspnoea, abdominal bloating, and oedema resolved completely.

**Figure 4:**
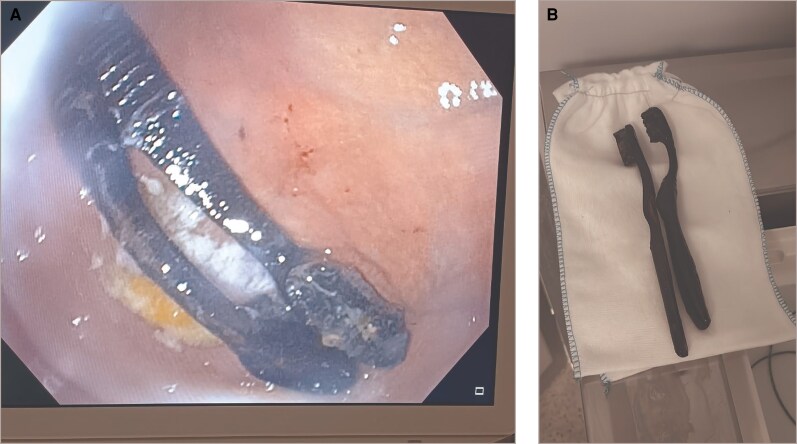
Endoscopic and post-extraction images. (*A*) Endoscopic view of a retained toothbrush within the stomach. (*B*) Photograph of the two extracted toothbrushes after endoscopic removal.

Psychiatric assessment found no evidence of psychosis or suicidal intent. The patient reported complete dentures because of early-onset periodontitis, but she denied having swallowed the toothbrushes. Repeated efforts to clarify the behavioural context were unsuccessful; at 1-month follow-up and again at 3-month review, she continued to state that she was surprised by the finding and did not know how the toothbrushes had come to be present.

At 1-month follow-up in the day hospital, NT-proBNP was 73 pg/mL (upper limit of normal <125 pg/mL). Repeat transthoracic echocardiography showed normal chamber dimensions (left ventricular end-diastolic diameter 4.3 cm; left ventricular end-systolic diameter 2.4 cm; right ventricular diameter 2.2 cm; left atrial diameter 3.3 cm; aortic root 2.3 cm), normal wall thickness (interventricular septum 1.0 cm; posterior wall 1.0 cm), preserved biventricular systolic function (left ventricular ejection fraction approximately 70%; TAPSE 24 mm), and a LAVI of 26 mL/m^2^. Diastolic indices were no longer suggestive of elevated filling pressures (E velocity 1.0 m/s, A velocity 0.54 m/s, deceleration time 141 ms, average E/e′ 7.22). The aortic valve was tricuspid, without stenosis or regurgitation. Mild tricuspid regurgitation persisted, with estimated RVSP up to 30 mmHg. No haemodynamically significant intracardiac shunt was seen. She remained asymptomatic. Representative one-month follow-up echocardiographic still frames and Doppler/tissue Doppler tracings are shown in *Figure 2E–H*.

At 3-month follow-up, the patient remained clinically well. Repeat transthoracic echocardiography was normal and NT-proBNP remained within the normal range (<125 pg/mL). She continued to deny having swallowed the toothbrushes and remained surprised by their presence despite repeated attempts to clarify this aspect of the history.

## Discussion

This case expands the clinical spectrum of gastro-cardiac syndrome by showing that retained gastric foreign bodies can present with acute heart-failure physiology rather than predominantly gastrointestinal symptoms. Previously published toothbrush-ingestion reports have focused mainly on endoscopic technique and gastrointestinal risk.^7–10^ In contrast, our patient presented with dyspnoea, oedema, elevated NT-proBNP, and echocardiographic findings interpreted as elevated filling pressures.

The toothbrushes themselves could not plausibly account for a 6-kg increase in body mass. The rapid weight gain was most consistent with reversible congestion and fluid retention, supported by peripheral oedema, elevated NT-proBNP, restrictive filling physiology on admission, and brisk post-extraction diuresis. A plausible mechanism is gastric distension leading to diaphragmatic elevation, altered intrathoracic pressure, impaired venous return, and possibly transient atrial compression, with additional autonomic effects mediated by vagal reflexes.^1–4^

Cardiovascular magnetic resonance was especially informative in this case. Although it excluded intrinsic myocardial or pericardial disease, its key contribution was the incidental identification of the gastric foreign bodies, which redirected the diagnostic evaluation towards an extracardiac cause. This diagnostic pivot deserves emphasis because it was the major reason the clinically relevant trigger was recognized. Representative high-resolution CMR images are now provided as a separate figure (*Figure 3*).

The follow-up findings further support reversibility: at 1-month follow-up, NT-proBNP had fallen to 73 pg/mL and the previously abnormal haemodynamic profile had normalized on echocardiography. Representative admission echocardiographic images/tracings and corresponding 1-month follow-up images/tracings are now provided together in *Figure 2*. At 3-month follow-up, echocardiography remained normal and NT-proBNP remained within the normal range. Finally, this case reinforces guideline-based management of foreign-body ingestion: long rigid objects such as toothbrushes are unlikely to pass spontaneously and should be removed urgently by endoscopy whenever feasible.^5,6^

## Data Availability

The data underlying this article are included in the article. Additional clinical details can be made available by the corresponding author on reasonable request, subject to patient confidentiality.

## Lead author biography

Ivana Jurin, MD, PhD, FESC is a cardiology consultant at Dubrava University Hospital, Zagreb, Croatia. She completed internal medicine training (2007–2012), a cardiology fellowship (2012–2017), and earned a PhD at the University of Zagreb School of Medicine (2019). Her interests include heart failure, acute coronary syndromes, atrial fibrillation and anticoagulation, pulmonary embolism, dyslipidaemia, valvular heart disease and transcatheter interventions, real-world registry research, and artificial intelligence in cardiology. She is a key investigator in the CaRD prospective registry and has participated in multicentre trials (VICTORION-2 Prevent, LANDMARK, ARO-APOC3-3001). She is a member of EACVI and EAPCI. She speaks Croatian and English.

## Notes

**Consent:** The authors confirm that written consent for submission and publication of this case report, including images and associated text, has been obtained from the patient, in line with COPE guidance.
